# Supplementary material for: The effect of different COVID-19 public health restrictions on mobility: A systematic review
Source: PLoS One. 2021 Dec 8;16(12):e0260919. doi: 10.1371/journal.pone.0260919 (PMC8654173; doi:10.1371/journal.pone.0260919)
Supplement: S1 File — (DOCX) [file pone.0260919.s002.docx]

**COVID-19 Living Evidence Database Search**

(“Google” or “Apple” AND “Mobility”)

OR

(global positioning)
