# Supplementary material for: The effect of different COVID-19 public health restrictions on mobility: A systematic review
Source: PLoS One. 2021 Dec 8;16(12):e0260919. doi: 10.1371/journal.pone.0260919 (PMC8654173; doi:10.1371/journal.pone.0260919)
Supplement: Attachment — Submitted filename: Comments.pdf [file pone.0260919.s004.pdf]

Summary of the manuscript: The authors reported a systematic review on the effect of different COVID-19 public health restrictions on mobility during the first wave of the pandemic. They found human mobility was significantly impacted because of imposed public restrictions particularly stay at home orders. They analyzed the effects of several interventions on mobility changes by analyzing Google mobility reports. These data are reported on six categories of places such as retail and recreation, groceries and pharmacies, parks, transit stations, workplaces, and residential. This review primarily focuses on data obtained from the first wave of the pandemic.
Although this is a good review however as the authors rightly pointed out that there are limitations with the Google Mobility data itself. I strongly believe that this Google Mobility data doesn't represent the accurate picture.

Comments and suggestions to the authors:

1. Line 41 to 44: the author says most studies analyzing data during the first two month i.e January and February, of pandemic.
   And during this time not all country introduced stringent restrictions to human movements. So how is this study not limited to low set of data.

2. Line 84 to 90: To facilitate surveillance of the public response to these restrictions, Google have released regular mobility reports. These anonymously report on changes in human mobility at a national or a local level.
   I am wondering how exhaustive these data are. This is because as compared to big nations like US, UN or Canada large chunks of small and underdeveloped countries don't have a good access of internet. Also, some countries don't use google at all. Then does this means google is not considering them pocket of populations? It would be nice if the author can comment on this.

3. Line 108 to 109: The reference lists of included articles were hand-searched to identify other potentially eligible studies for inclusion in the analysis missed by the initial search or any unpublished data.
   Can you elaborate what do you mean by hand-search. What are the criteria for identifying the potential study using this method?

4. Line 159 to 160: From the initial search, 1672 references were identified, of which 85 were selected for full text checking (Figure 1). From these 71 were excluded and 14 were included in the narrative synthesis (Figure 1).
   It's a very nice flowchart, however it's not clear to me the rationale of screening and eligibility. It would be nice if the author includes few lines describing the criteria, they have used to screen studies for their narrative synthesis.

5. Line 380 to 382: Given the individual, societal and environmental benefits of public transport use, there is a need to plan for how encourage people back on to public transport, supporting social distancing and rebuilding confidence
   Correct the typo between line 380-381.

6. Line 380 to 382: A limitation of the included studies was that they reported data exclusively from the first wave of the pandemic.
   Did first wave happened at the same time in all the countries of the world? Can you specify the time to provide more clarity to the readers?

7. Line 387 to 388: Warnings have been given that populations would not be able to sustain transmission prevention behaviours, such as reduced mobility.
   Can you provide a reference for this?

8. Line 390 to 392: A cross-sectional study of adults in Australia, USA and UK showed that although adherence was restrictions remained high into the second wave, it did decline, particularly in young people and males.
   Correct the typo in line 391.

9. Line 491: Summan A, Nandi A. Timing of non-pharmaceutical interventions to mitigate COVID-19 transmission and their effects on mobility: A cross-country analysis. medRxiv 2020.05.09.2009642.
   Please change the DOI of the reference.
